# Can Elevated Pretreatment Serum Carcinoembryonic Antigen Levels Serve as a Potential Biomarker Guiding Adjuvant Chemotherapy in Rectal Cancer Patients With ypTis-3N0 After Neoadjuvant Radiotherapy and Surgery?

**DOI:** 10.3389/fonc.2021.705460

**Published:** 2021-08-03

**Authors:** Chi Huang, Mingkun Jiang, Yan Li, Chaoyang Tang, Xiang Ma, Xiangkun Huan

**Affiliations:** ^1^Department of General Surgery, Affiliated Hospital of Integrated Chinese and Western Medicine, Nanjing University of Chinese Medicine, Nanjing, China; ^2^Department of General Surgery, The Second Affiliated Hospital of Nanjing Medical University, Nanjing, China; ^3^Department of General Surgery, Yinchuan Second People’s Hospital, Yinchuan, China; ^4^Department of Surgical Oncology, Jiangsu Province Hospital of Chinese Medicine, Affiliated Hospital of Nanjing University of Chinese Medicine, Nanjing, China

**Keywords:** neoadjuvant radiotherapy, rectal cancer, serum carcinoembryonic antigen, ypTis-3N0, surgery

## Abstract

Survival benefit of adjuvant chemotherapy (ACT) remained controversial in patients with stage II/III rectal cancer (RC) who received neoadjuvant therapy and surgery. This study aimed to investigate the guiding role of elevated pretreatment serum carcinoembryonic antigen (CEA) levels for receiving ACT in yield pathological Tis-3N0 (ypTis-3N0) RC patients after neoadjuvant radiotherapy and surgery. Between 2004 and 2015, 10,973 RC patients with ypTis-3N0 who received neoadjuvant radiotherapy and radical surgery were retrospectively analyzed using the Surveillance, Epidemiology, and End Results (SEER) database. Compared with CEA-normal group, elevated-CEA patients had worse 5-year CSS rate (90.1 *vs* 83.5%). The 5-year CSS rates were 86.3 and 87.4% for ypTis-3N0M0 patients with or without ACT, respectively. Patients receiving ACT had a comparable 5-year CSS rate compared to those who did not regardless of CEA levels in ypTis-3N0M0 RC patients (CEA elevation group: 76.4 *vs*. 83.5%, P = 0.305; CEA normal group: 90.0 *vs*. 90.1%, P = 0.943). Intriguingly, ypT3N0M0 RC patients with elevated CEA levels may benefit from ACT (5-year CSS: 69.1 *vs*. 82.9%, P = 0.045), while those with normal CEA levels did not (5-year CSS: 89.3 *vs*. 89.3%, P = 0.885). Multivariate Cox analysis demonstrated that ACT tended to be a protective factor in elevated-CEA ypT3N0M0 RC patients (HR = 0.633, 95% CI = 0.344–1.164, P = 0.141), while ACT was not associated with improved CSS in normal-CEA ypT3N0M0 RC patients (HR = 1.035, 95% CI = 0.487–2.202, P = 0.928). Elevated pretreatment serum CEA levels may serve as a promising biomarker guiding ACT in rectal cancer patients with ypT3N0M0.

## Introduction

Based on the results from the German Rectal Cancer Study Group (the CAO/ARO/AIO-94 trial) that demonstrated preoperative chemoradiotherapy (CRT) could decrease local recurrence among patients with locally advanced rectal cancer compared to postoperative chemoradiotherapy (1, 2), neoadjuvant CRT followed by radical resection has been established as a standard strategy for locally advanced rectal cancer.

Satisfactory regression has often been observed after neoadjuvant radiotherapy (RT), and some patients even achieved clinical complete response (CCR) or pathological complete response (PCR), which brings debates to the choice of adjuvant chemotherapy (ACT) (3). ACT could reduce the risk of recurrence and mortality for patients with locally advanced rectal cancer (4). However, ACT could also bring systemic toxicity problems. Conclusive data on the use of ACT depending on pretreatment clinical stage or yield pathological stage are lacking. Patients with rectal cancer were often excluded from phase III studies due to the potential impact of RT or CRT. For colon cancer, survival benefit of ACT has been observed for patients with ‘high-risk’ stage II and stage III disease (5). According to yield pathological stage, ACT will no longer be needed in patients with ypTis-2N0 and “low-risk” ypT3N0. Besides, it is hard to determine real ‘high-risk’ stage II after preoperative CRT. Evidence from some studies indicated that patients with pathological complete response (pCR) did not benefit from ACT (6, 7), while other studies had come to the opposite conclusion (8, 9). However, the National Comprehensive Cancer Network (NCCN) recommended use of ACT for patients with stage II/III rectal cancer regardless of postoperative yield pathology if the patient did not receive neoadjuvant chemotherapy. The European Society for Medical Oncology (ESMO) indicated that it was reasonable to consider ACT in rectal cancer patients after preoperative chemoradiotherapy with yp stage III and “high-risk” yp stage II. In fact, the role of ACT in patients after neoadjuvant CRT and surgery has not been well established.

Previous studies reported the use of serum carcinoembryonic antigen (CEA) levels to guide ACT for stage IIA colon cancer (10). Patients with elevated pretreatment CEA levels should be grouped into ‘high-risk’ stage II disease. Inspired from these points of view, we have evaluated the guiding role of elevated pretreatment serum CEA levels for use of ACT in ypTis-3N0 rectal cancer using the Surveillance, Epidemiology, and End Results (SEER) database.

## Methods

### Patient Selection

Patients with ypTis-3N0 rectal cancer who received neoadjuvant radiotherapy and underwent definitive/curative surgery were included and retrospectively analyzed from the SEER database (2004–2015): pretreatment serum CEA information was available starting from 2004. This study was approved by the Institutional Review Board of Affiliated Hospital of Nanjing University of Chinese Medicine. The inclusion criteria were listed as follows: the site code represented “rectum (130)”; patients received ‘‘radiation before surgery’’ (2, preoperative radiotherapy); surgery was performed in primary site; patients with ypTis-3N0M0; information about cancer-specific survival (CSS), and survival months were available. All patients were enrolled in the current analysis according to the American Joint Committee on Cancer staging system. Preoperative radiotherapy is mainly beam radiotherapy, and a few patients used radioactive implants or radioisotopes; the main methods of operation were abdomen perineal reservation (APR) and anterior resection (AR), and the specific chemotherapy regimen was unknown; according to the SEER database.

### Data Collection

The following data were gathered: gender, age at diagnosis, marital status, race, tumor size, T stage, histologic type, differentiation status, pretreatment serum CEA levels, CSS, and survival months. CSS represented the time from the date of initial diagnosis to the date of death resulting from rectal cancer. Among them, the age should be over 18 years old, rectal cancer was primary, and the cut-off value of CEA level was selected as 5 ng/ml. It should be noted that the study lacked data on complications in patients with rectal cancer, which is an important factor for survival.

### Statistical Analysis

The differences between two groups (the CEA-normal group and CEA-elevated group regardless of whether having received ACT, the receiving ACT group and not receiving ACT group regardless of the level of CEA, the receiving ACT group and not receiving ACT group in condition of CEA-elevated and CEA-normal, respectively.) were compared using χ2 test. The Kaplan–Meier method was adopted to evaluate CSS and to estimate relative 5-year survival rate. The difference was compared with log-rank test. Cox proportional hazards regression models were performed to screen out independent factors which were associated with CSS. To minimize the risk of biased estimates of treatment effect, propensity score matching (PSM) at a 1:2 ratio was performed. The PSM model included gender, age, marital status, race, tumor size, T stage, histologic type, and differentiation status. All statistical analyses were performed with SPSS 25.0 and R (version 3.6.0).

## Results

### Pretreatment Serum CEA Levels Is an Independent Prognostic Factor in ypTis-3N0M0 Rectal Cancer

**Figure 1:**
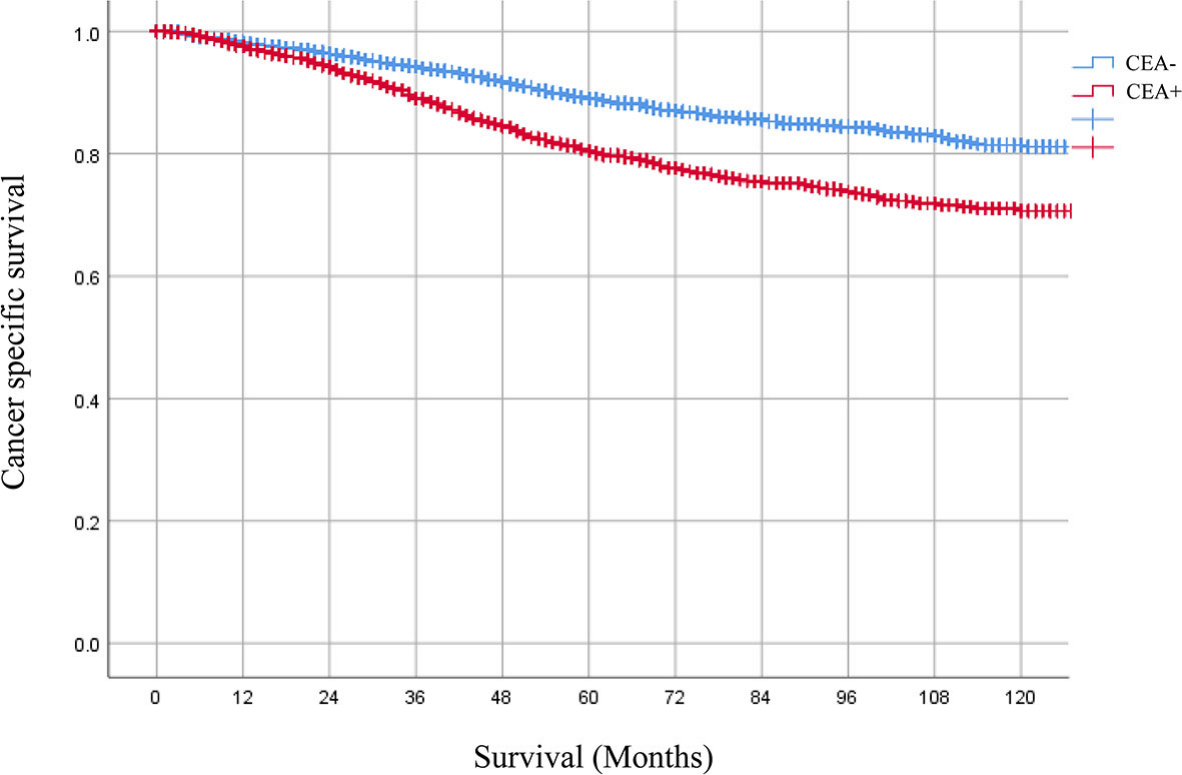
Kaplan–Meier CSS curves of patients with elevated or normal pretreatment serum. CEA levels were 90.1 and 83.5%, respectively. HR = 1.597, 95% CI = 1.385–1.841, P < 0.001.

A total of 6,806 ypTis-3N0M0 rectal cancer patients with known pretreatment serum CEA levels were identified from the SEER database. Among them, 4,190 patients were grouped into the CEA-normal group, 2,616 patients were grouped into the CEA-elevated group. Compared with the CEA-normal group, patients with elevated pretreatment serum CEA levels had worse 5-year cancer-specific survival (CSS) rate (90.1 *vs*. 83.5%) (**Figure 1**). Multivariate Cox analysis demonstrated that elevated pretreatment serum CEA level was an independent risk factor in the cohort (HR = 1.597, 95% CI = 1.385–1.841, P < 0.001) (**Table 1**). Intriguingly, multivariate Cox analyses showed that pretreatment serum CEA elevation in stage ypTis-1N0M0 group presented the most remarkable increased risk of CSS compared with stage ypT2N0M0 or ypT3N0M0 group (ypTis-1N0M0: HR = 1.891, 95% CI = 1.286–2.781, P = 0.001; ypT2N0M0: HR = 1.465, 95% CI = 1.008–2.129, P = 0.045; ypT3N0M0: HR = 1.570, 95% CI = 1.325–1.861, P < 0.001) (**Table 2**).

**Table 1:** Multivariate Cox regression analyses of CSS in ypTis-3N0M0 rectal cancer patients with pretreatment serum CEA level.

Covariate	Reference	Characteristic	Cancer-specific survival		
			HR(95%CI)	SE	P value
Age (year)	≤60	>60	1.323 (1.145–1.527)	0.073	<0.001*
Race	White	Black	1.214 (0.964–1.528)	0.117	0.099
		Other	0.785 (0.605–1.020)	0.133	0.070
Marital status	Unmarried	Married	0.727 (0.627–0.843)	0.075	<0.001*
		Unknown	0.589 (0.345–1.007)	0.273	0.053
Gender	Male	Female	0.814 (0.699–0.947)	0.078	0.008*
Grade	G1 + G2	G + G4	1.565 (1.341–1.828)	0.079	<0.001*
		Unknown	0.787 (0.657–0.943)	0.092	0.009*
Histology	Adenocarcinoma	Mucinous adenocarcinoma	1.362 (1.034–1.794)	0.140	0.028*
		Signet ring cell carcinoma	6.202 (3.519–10.928)	0.289	<0.001*
		Other	1.114 (0.902–1.376)	0.108	0.314
Tumor size	<5.0 cm	≥5.0 cm	1.161 (0.979–1.377)	0.087	0.087
		Unknown	1.169 (1.983–1.390)	0.088	0.078
CEA level	Normal	Elevated	1.597 (1.385–1.841)	0.073	<0.001*

*P < 0.05 was considered significant.

**Table 2:** Multivariate Cox regression analyses of the role of pretreatment serum CEA level on CSS in patients with different ypT stage.

ypT stage	Reference	Characteristic	Cancer-specific survival		
			HR(95%CI)	SE	P value
ypTis-1	Normal	Elevated	1.891 (1.286–2.781)	0.197	0.001*
ypT2			1.465 (1.008–2.129)	0.191	0.045*
ypT3			1.570 (1.325–1.861)	0.087	<0.001*

*P < 0.05 was considered significant.

### ACT Was Not Associated With Improved CSS in Patients With ypTis-3N0M0 Rectal Cancer

**Figure 2:**
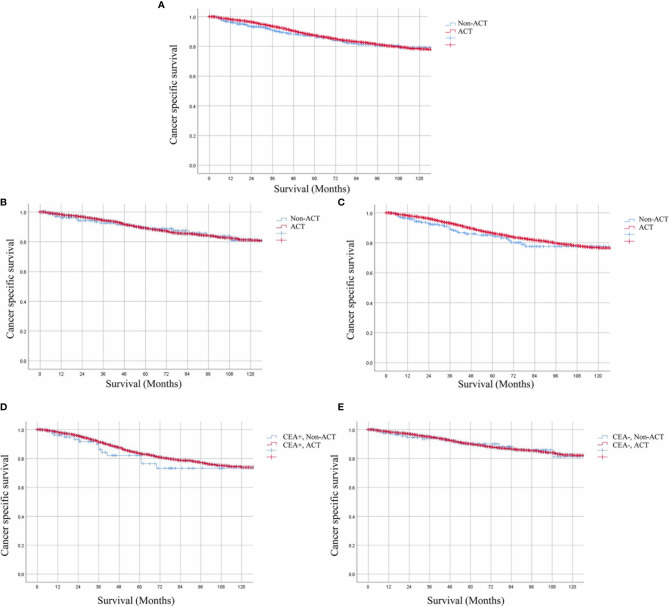
Kaplan–Meier CSS curves of patients receiving or not receiving ACT. **(A)** The 5-year CSS estimates were 86.3 and 87.4% for ypTis-3N0M0 rectal cancer patients with ACT and without ACT. **(B, C)** Patients with ypTis-2N0M0 (HR = 1.012, 95% CI = 0.638–1.606, P = 0.958.) or ypT3N0M0 (HR = 0.906, 95% CI = 0.632–1.298, P = 0.590). Rectal cancer may not benefit from ACT. **(D, E)**. Kaplan–Meier analysis demonstrated that the 5-year CSS rate of ypTis-3N0M0 rectal cancer patients receiving ACT and those without receiving ACT were 76.4 and 83.5% (HR = 0.830, 95% CI = 0.484–1.422, P = 0.497), respectively in the setting of elevated pretreatment serum CEA levels; 90.0 and 90.1% (HR = 0.966, 95% CI = 0.554–1.684, P = 0.904), respectively, in the setting of normal pretreatment serum CEA levels.

A total of 10,973 patients with ypTis-3N0M0 rectal cancer were identified from the SEER database. Among them, 10,594 patients received ACT, 379 patients did not receive ACT. Kaplan–Meier survival curves revealed that patients with ypTis-3N0M0 rectal cancer may not benefit from ACT (**Figure 2A**). The 5-year CSS estimates were 86.3 and 87.4 for patients with ACT and without ACT, respectively. Multivariate Cox analysis demonstrated that ACT was not associated with improved CSS in patients with ypTis-3N0M0 rectal cancer (HR = 0.971, 95% CI = 0.731–1.288, P = 0.836). Subgroup analysis revealed that patients with ypTis-2N0M0 or ypT3N0M0 rectal cancer may not benefit from ACT (**Figures 2B, C**). Similarly, multivariate Cox analysis also revealed that ACT was not associated with improved CSS in patients with ypTis-2N0M0 or ypT3N0M0 rectal cancer (ypTis-2N0M0: HR = 1.012, 95% CI = 0.638–1.606, P = 0.958; ypT3N0M0: HR = 0.906, 95% CI = 0.632–1.298, P = 0.590).

### Evaluating Associations of the Pretreatment Serum CEA Levels and ACT on the Basis of CSS

**Figure 3:**
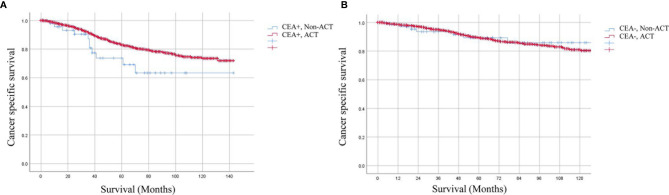
Kaplan–Meier CSS curves stratified by the combination of pretreatment serum CEA levels and receiving ACT in different stages. **(A)** The 5-year CSS rates of ypT3N0M0 rectal cancer patients with elevated pretreatment serum CEA levels were 69.1 and 82.9% (HR = 0.633, 95% CI = 0.344–1.164, P = 0.141), respectively, **(B)** while ypT3N0M0 rectal cancer patients with normal pretreatment serum CEA levels were 89.3 and 89.3% (HR = 1.035, 95% CI = 0.487–2.202, P = 0.928), respectively.

For ypTis-3N0M0 rectal cancer patients, Kaplan–Meier analysis demonstrated that patients receiving ACT had comparable 5-year CSS rate as compared to those not receiving ACT in the setting of elevated pretreatment serum CEA levels (76.4 *vs* 83.5%, P = 0.305) (**Figure 2D**). In the setting of normal pretreatment serum CEA levels, 5-year CSS rate of patients receiving ACT was similar to those not receiving ACT (90.0 *vs*. 90.1%, P = 0.943) (**Figure 2E**). Multivariate Cox analysis also revealed that ACT was not associated with improved CSS regardless of pretreatment serum CEA levels in ypTis-3N0M0 rectal cancer patients (elevated-CEA group: HR = 0.830, 95% CI = 0.484–1.422, P = 0.497; CEA normal group: HR = 0.966, 95% CI = 0.554–1.684, P = 0.904). Intriguingly, ypT3N0M0 rectal cancer patients with elevated pretreatment serum CEA levels may benefit from ACT (5-year CSS: 69.1 *vs*. 82.9%, P = 0.045) (**Figure 3A**), while ypT3N0M0 rectal cancer patients with normal pretreatment serum CEA levels did not benefit from ACT (5-year CSS: 89.3 *vs*. 89.3%, P = 0.885) (**Figure 3B**). Multivariate Cox analysis demonstrated that ACT tended to be a protective factor in ypT3N0M0 rectal cancer patients with elevated pretreatment serum CEA levels (HR = 0.633, 95% CI = 0.344–1.164, P = 0.141), while ACT was not associated with improved CSS in ypT3N0M0 rectal cancer patients with normal pretreatment serum CEA levels (HR = 1.035, 95% CI = 0.487–2.202, P = 0.928).

### CSS of ACT in ypT3N0M0 Rectal Cancer Patients With Elevated Serum CEA Levels After PSM

**Figure 4:**
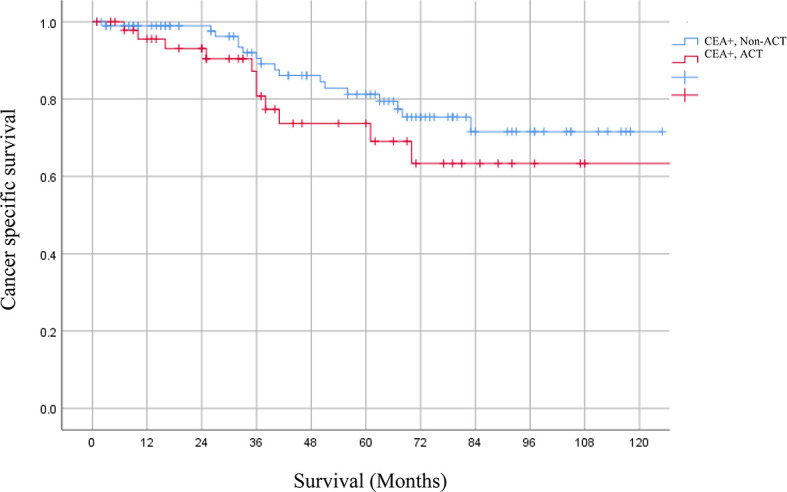
The 5-year CSS rates of ypT3N0M0 rectal cancer patients with elevated serum CEA levels receiving or not receiving ACT after PSM were 69.1 and 77.4% (P = 0.216), respectively.

After PSM, 147 ypT3N0M0 rectal cancer patients with elevated serum CEA levels were involved. 98 patients received ACT and 49 patients did not receive ACT; no characteristics showed statistical differences between the two groups. However, Kaplan–Meier analysis revealed that patients receiving ACT had comparable 5-year CSS rate as compared to those without receiving ACT (69.1 *vs*. 77.4%, P = 0.216) (**Figure 4**).

## Discussion

Rectal cancer is a malignant tumor that originates in the epithelium of the rectal mucosa. It is asymptomatic in the early stage and has stool characteristics and changes in bowel habits in the late stage. According to the American Joint Committee on Cancer (AJCC)/International Union Against Cancer (UICC) eighth edition colorectal cancer TNM staging system, rectal cancer can be divided into stages 0–IV according to the severity. The treatment of rectal cancer is a comprehensive treatment based on surgery, including chemotherapy and radiotherapy. Surgical methods include classic Miles surgery, Dixon surgery, *etc.*, which specifically refer to NCCN rectal cancer treatment guidelines (11).

Before neoadjuvant radiotherapy had been adopted as a routine clinical practice in locally advanced middle and low rectal cancer, several studies demonstrated that ACT could improve the prognosis of patients with Dukes’ B and Dukes’ C stages (12). A systematic review including 21 eligible randomized controlled trials (RCTs) showed a reduction in the risk of mortality (17%) and disease recurrence (25%) of ACT in rectal cancer (13). However, two limitations need attention. The patients who received neoadjuvant radiotherapy were enrolled in only two RCTs. No modern drugs, such as oxaliplatin, were included in the ACT. The adoption of ACT largely depended on pathological TNM stage. Neoadjuvant radiotherapy resulted in tumor down-sizing and down-staging; some patients (ypTis-3N0M0) no longer needed ACT according to previous criteria. However, clinicians would prefer to adopt ACT in clinical practice despite the lack of high-level evidence.

The EORTC 22921 study randomly assigned patients with clinical stage T3 or T4 resectable rectal cancer to receive preoperative radiotherapy with or without concomitant chemotherapy before surgery followed by either ACT or surveillance. With regret, ACT after preoperative radiotherapy was not associated with improved DFS or OS after a median follow-up of 10.4 years (14). Similarly, another three trials did not classify the value of ACT (15–17). Based on the four trials, a systematic review and meta-analysis yielded the same results (18). However, the limitations of the above trials are obvious. The major problem was poor adherence to ACT. The value of ACT may be partially impaired. Evidence from ADORE trial indicated that adjuvant FOLFOX was associated with improved DFS compared with fluorouracil plus leucovorin in patients with locally advanced rectal cancer after neoadjuvant chemoradiotherapy and surgery (19). At present, there are still disputes about the value of ACT for locally advanced rectal cancer patients who received neoadjuvant CRT and surgery.

Serum CEA is the most important tumor marker for the presence of subclinical hepatic or pulmonary metastases, and elevated pretreatment serum CEA levels were significantly associated with poor prognosis in rectal cancer patients (20, 21). Besides, serum CEA levels could predict PCR after neoadjuvant therapy for rectal cancer (22). The American Joint Committee on Cancer (AJCC) had suggested that serum CEA levels serve as an additional factor for clinical care. Combination of carcinoembryonic antigen with the AJCC TNM staging system could improve prognostic precision for rectal cancer (23). Recently, several studies have reported the use of serum CEA levels to guide ACT in stage II colon cancer patients (10, 24, 25). However, another study found that stage IIA colon cancer patients with elevated pretreatment serum CEA levels did not show survival benefit from ACT. Can elevated pretreatment serum carcinoembryonic antigen levels guide ACT in rectal cancer patients with ypTis-3N0 after neoadjuvant radiotherapy and surgery? No previous studies explored the predictive value of pretreatment serum CEA levels to adaptation of ACT in rectal cancer patients after neoadjuvant radiotherapy and surgery. For patients with yp T4 or yp stage III, adoption of ACT is well-accepted, while the value of ACT in patients with ypTis-3N0 is full of controversy.

In the present study, we first found that pretreatment serum CEA levels were an independent prognostic factor in ypTis-3N0M0 rectal cancer. Intriguingly, multivariate Cox analyses showed that pretreatment serum CEA elevation in stage ypTis-1N0M0 group presented the most remarkable increased risk of CSS compared with stage ypT2N0M0 or ypT3N0M0 group. Early stage rectal cancer with elevated serum CEA levels presented with more aggressive behavior and unexpected poor prognosis. This subgroup needed more intensive follow-up and intervention.

To the best of our knowledge, this is the first study to investigate the value of pretreatment serum CEA levels for guiding ACT in rectal cancer patients with ypTis-3N0 after neoadjuvant radiotherapy and surgery. To evaluate the value of ACT, multivariate Cox analysis demonstrated that patients with ypTis-3N0M0 rectal cancer did not benefit from ACT. Further, we evaluated associations of the pretreatment serum CEA levels and ACT on the basis of CSS. Similarly, ACT was not associated with improved CSS regardless of pretreatment serum CEA levels in ypTis-3N0M0 rectal cancer patients. However, ypT3N0M0 rectal cancer patients with elevated pretreatment serum CEA levels who received ACT had superior 5-year CSS than those who did not receive ACT, while ypT3N0M0 rectal cancer patients with normal pretreatment serum CEA levels did not benefit from ACT. Although multivariate Cox analysis did not confirm the value of ACT in ypT3N0M0 rectal cancer patients with elevated pretreatment serum CEA levels, a trend toward a protective factor of ACT was observed. A relatively small sample size may result in insufficient power in our study. Especially, a large cohort is needed to verify the value of pretreatment serum CEA levels for guiding ACT in rectal cancer patients with ypT3N0M0. The results will have a profound effect on clinical practice.

Several limitations are inevitable in our present study. First, the lack of serum CEA levels after neoadjuvant CRT made it impossible to compare with pretreatment serum CEA levels, resulting in insufficient evaluation of the value of serum CEA levels. Second, the SEER database did not include other important prognostic factors, such as the regime and course of chemotherapy, and the adherence to ACT. Third, clinical staging, which is indispensable for selection of neoadjuvant CRT, was unavailable.

In conclusion, elevated pretreatment serum carcinoembryonic antigen levels may serve as a promising biomarker guiding ACT in rectal cancer patients with stage ypT3N0M0. Further study with larger sample size is needed to verify our results.

## Data Availability Statement

The datasets are available in the SEER repository and can be obtained from https://seer.cancer.gov.

## Author Contributions

XH and XM conceived this study. CH and MJ improved the study design and contributed to the interpretation of results. YL collected the data. CT performed data processing and statistical analysis. XH and XM wrote the manuscript. CH and MJ revised the manuscript. All authors contributed to the article and approved the submitted version.

## Funding

General projects of natural science research projects in Universities of Jiangsu Province (20KJB320015); The Science and Technology Development Fund of Nanjing Medical University (NMUB2019049); General Project of Nanjing Medical Technology Development Fund (YKK18192); The Key R&D Program of Jiangsu Province (Social Development, BE2019768); The Programs of Jiangsu Province Hospital of Chinese Medicine (Y2019CX43).

## Conflict of Interest

The authors declare that the research was conducted in the absence of any commercial or financial relationships that could be construed as a potential conflict of interest.

## Publisher’s Note

All claims expressed in this article are solely those of the authors and do not necessarily represent those of their affiliated organizations, or those of the publisher, the editors and the reviewers. Any product that may be evaluated in this article, or claim that may be made by its manufacturer, is not guaranteed or endorsed by the publisher.
